# Supplementary material for: Adapting Elements of Cleft Care Protocols in Low- and Middle-income Countries During and After COVID-19: A Process-driven Review With Recommendations
Source: Cleft Palate Craniofac J. 2022 Jan 4;60(5):526–35. doi: 10.1177/10556656211069827 (PMC10102820; doi:10.1177/10556656211069827)
Supplement: sj-docx-1-cpc-10.1177_10556656211069827 - Supplemental material for Adapting Elements of Cleft Care Protocols in Low- and Middle-income Countries During and After COVID-19: A Process-driven Review With Recommendations [file sj-docx-1-cpc-10.1177_10556656211069827.docx]

**Supplementary data**

**Supplementary Figure 1:** Circle of Cleft Professionals Questionnaire and results


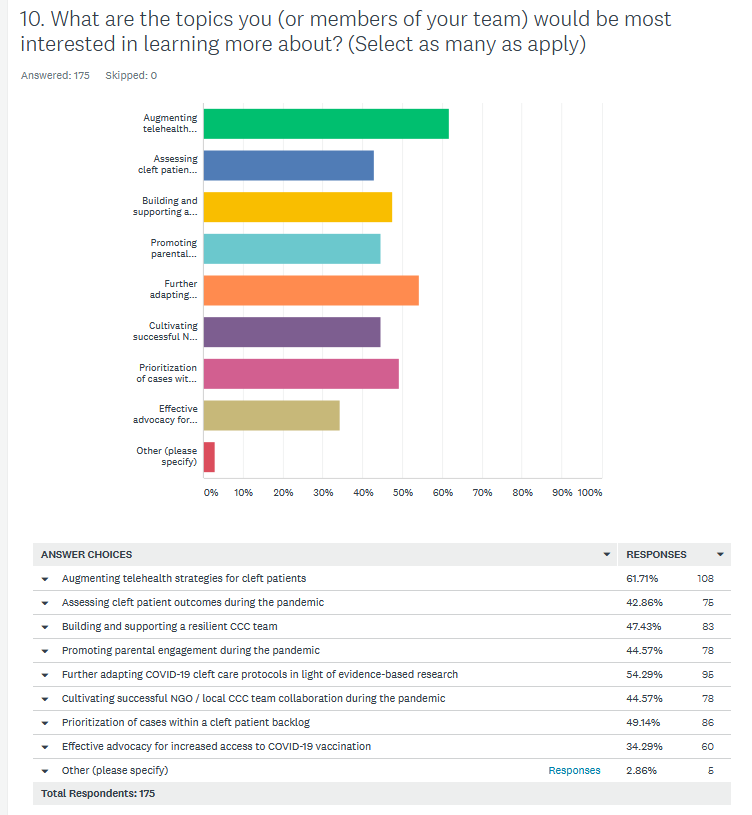


**Supplementary Table 1:** Evidence that timing of palatoplasty is associated with speech outcome

| **Resource title** | **Level of Evidence** | **Overview of resource** | **Conclusion** |
| --- | --- | --- | --- |
| SCANDCLEFT Trials  (Lohmander et al., 2017)    (Willadsen et al., 2017) | 1 | RCT comparing 4 2-stage surgical protocols for complete UCLP  Hard palate closure at 12 versus 36 months was compared | No difference in resonance (nasality)  Later palate closure associated with poorer consonant proficiency |
| (Willadsen et al., 2018)  Denmark | 1 | RCT 126 children with UCLP to compare hard palate closure at 12 months versus 36 months | Early hard palate closure associated with better consonant production and fewer active cleft speech characteristic errors |
| (Willadsen, 2012)  Denmark | 1 | RCT 34 children with UCLP  SP closure at 4 months, HP closure at 12 months or 36 months | Speech outcomes better in early repair group – 12 months. |
| (Williams et al., 2011)  Brazil | 1 | RCT comparing primary palate closure at 9-12 months versus 15-18 months | No statistical difference in resonance |
| (Shaffer et al., 2020) | 4 | Retrospective review 733 children born 2005-15  Age at palatoplasty: Early: <11mnths, n=28;  Standard: 11-13mnths, n=158; Late: >13mnths, n=46 | Late palatoplasty associated with increased speech and language delay.  Speech sound production disorders, VPI and hearing loss were not significantly associated with age at palatoplasty |
| (Kara et al., 2020)  Turkey | 4 | Retrospective review of 416 cleft palate patients who had palate repair before 12 months, 12-18 months and >18 months | Palate repairs after 18 months had worse speech |
| (Swanson et al., 2017) | 4 | Retrospective review of 40 patients with submucous palate | Better speech outcomes in patients repaired before 4 years of age |
| (Follmar et al., 2015) | 4 | 13yr retrospective review of 201 internationally adopted patients 1993-2006. 183 surgery before 18 months, 18 performed after 18 months.  33% of delayed group developed VPI compared 13% of those repaired at <18 months (p=0.03) | Internationally adopted children whose palate was repaired after 18 months were more likely to develop speech symptoms of VPI. |
| (Pasick et al., 2014) | 4 | Retrospective review of 24 patients : palatoplasty at >18 months | Palate repair after 18/12 associated with significantly increased incidence of articulation errors associated with VPI |
| (Yang et al., 2013) | 4 | Retrospective review of 503 patients with non-syndromic cleft palate | Repair before 2 years of age had better velopharyngeal competence |
| (Chapman et al., 2008) | 4 | 40 children with non-syndromic cleft palate repaired primarily in younger age (mean 11 months) and older age (mean 15 months) assessed at age 3 years. | At age 3 years children who had palate surgery prior to the onset of words had better articulation and resonance outcomes than those who had surgery after the onset of words. |
| (Hardin-Jones & Jones, 2005) | 4 | 212 preschoolers with clefts | Significant relationship between age at palatal surgery and prevalence of hypernasality advocated primary repair before 13 months |
| (Rezaei et al., 2020)  Iran | 4 | Retrospective review of 180 patients – comparison made between primary palate repair before and after 13 months | Late surgery group had significantly worse articulation errors and more likely to have moderate/severe hypernasality |
| CRANE Database UK  (CRANE, 2020) | 4 | Comparing speech outcomes with timing of surgery for 789 non-syndromic children born 2007-2013. Significant difference in speech outcome with those having surgery at 14+ months doing significantly worse on all 3 UK speech standard outcomes. | Speech outcomes better in children who have speech surgery before 14 months of age. |
| (Marrinan et al., 1998) | 4 | Retrospective study of 228 non-syndromic patients with 2 surgeons. Significant linear association (p=.025) between age at repair and VPI. | Patients in the early repair group (8-10 months) were least likely to need secondary treatment for VPI. |
| (Dorf & Curtin, 1982) | 4 | 21 children surgery before 12 months,  59 surgery after 12.5 months  10% in early group had compensatory articulations compared to 86% in late group | Worse articulation outcomes for children whose palate surgery was completed before 12 months compared to those completed after 12 months. |
| (Peterson-Falzone, 1996) | 5 | A non-systematic review of the evidence on timing of palate surgery and speech outcome | The effects of structural constraints on phonetic and phonological development in children supports efforts towards earlier surgery (<12 months).General trend in the literature of better speech results in earlier surgery |

**Supplementary Table 2:** Evidence that the timing of primary palatoplasty is associated with maxillary growth outcome

| **Study /Personal Experience** | **Level of Evidence** | **Overview** | **Conclusion** |
| --- | --- | --- | --- |
| (Pereira et al., 2018)  Brazil | 1 | Single centre RCT of 64 patients to evaluate early complete palate repair (9-15 months) vs late hard palate repair (3-4 years) | Delayed hard palate repair had better dentofacial growth |
| Scandcleft Trials  (Küseler et al., 2019)  (Heliövaara et al., 2017)  (Karsten et al., 2017) | 1 | Part of a multi-centre RCT of 429 patients compared early (12 months) versus late (36 months) hard palate repair. Maxillary growth measured using models at 5 years and cephalometry at 8 years | No difference in maxillary growth by 8 years of age |
| (Botticelli et al., 2019)  Denmark but included in Scandcleft | 1 | Single centre RCT of 122 UCLP to compare hard palate closure at 12 or 36 months – assessment on models at 8 years of age | No conclusion about which was favourable – delayed repair had better transverse dimension but had a shallower morphology |
| (Salgado et al., 2019) | 4 | A systematic review or early vs delayed palatoplasty and effect on growth – 5 included observational studies   - Daskalogiannakis et al., 2006 - Holland et al., 2007 - Yaminishi et al., 2011 - Zemann et al., 2011 - Bakri et al., 2014 | Conflicting results between the five studies – no conclusion made |
| (Xu et al., 2012) | 4 | Retrospective series of 46 UCLP to compare palatoplasty before and after 4 years of age | Better maxillary growth associated with later repairs |
| (Y.-F. Liao et al., 2010) | 4 | Retrospective series of 72 cUCLP to compare one stage versus two-stage with delayed hard palate repair. Cephalometry at 20 years. | Delayed hard palate associated with better maxillary growth |
| (Y.-F. Liao & Mars, 2006) | 3 | A systematic review of timing of palate repair on facial growth – 15 observational studies included | No conclusive evidence that timing affects growth |
| (Y. F. Liao et al., 2006) | 4 | Retrospective series of 104 patients in Sri Lanka who had their palate repaired by 13 years of age | Later age of repair was associated with better AP growth of the maxilla |
| (Friede, 2007) | 5 | Personal perspectives of delayed hard palate closure | Advocates delayed hard palate closure at 1-1.5 years to achieve better growth |

**Supplementary Table 3:** The Federation of Surgical Specialty Associations Surgical Prioritisation System – first published June 2020. Updated in February 2021. The priority levels assigned to cleft operations are indicated both initially in July 2020 and later in February 2021.

| **Priority** | **Time scale for surgical procedure to be performed** | **Initial cleft operation prioritisation July 2020** | **Amendment to cleft prioritisation February 2021** |
| --- | --- | --- | --- |
| 1a | Less than 24 hours | Nil | Nil |
| 1b | Less than 72 hours | Nil | Nil |
| 2 | Less than 1 month | Nil | Primary cleft palate repair (child breaching 13 months of age),  Secondary speech surgery (child breaching 5 years of age) |
| 3 | Less than 3 months | Primary Palatoplasty,  Secondary Speech Surgery  Alveolar Bone Grafting | Primary Palatoplasty (child less than 12 months of age),  Secondary Speech Surgery (child less than 5 years of age)  Alveolar Bone Grafting (prior to canine eruption) |
| 4 | More than 3 months | All other cleft operations | All other cleft operations |

**References**

Botticelli S, Küseler A, Mølsted K, Ovsenik M, Nørholt SE, Dalstra M, Cattaneo PM, Pedersen TK. Palatal morphology in unilateral cleft lip and palate patients: Association with infant cleft dimensions and timing of hard palate repair. *Orthod Craniofac Res*. 2019;*22*(4):270-280.

Chapman, KL, Hardin-Jones MA, Goldstein JA, Halter KA, Havlik RJ, Schulte J. Timing of palatal surgery and speech outcome. *Cleft Palate Craniofac J*. 2008;*45*(3):297–308.

CRANE. Crane Database Annual Report 2020. Available at: <https://www.crane-database.org.uk/reports/?filter_date=2020>. Accessed October 14, 2021.

Dorf DS, Curtin JW. Early cleft palate repair and speech outcome. *Plast Reconstr Surg*. 1982; 70(1):74-81.

Follmar KE, Yuan N, Pendleton CS, Dorafshar AH, Kolk CV, Redett RJ. Velopharyngeal Insufficiency Rates After Delayed Cleft Palate Repair. *Ann Plast Surg*. 2015;*75*(3):302-305.

Friede H. Maxillary Growth Controversies after Two-Stage Palatal Repair with Delayed Hard Palate Closure in Unilateral Cleft Lip and Palate Patients: Perspectives from Literature and Personal Experience. *Cleft Palate Craniofac J*. 2007;*44*(2):129-136.

Hardin-Jones MA, Jones DL. Speech Production of Preschoolers with Cleft Palate. *Cleft Palate Craniofac J*. 2005;*42*(1):7-13.

Heliövaara A, Küseler A, Skaare P, Shaw W, Mølsted K, Karsten A, Brinck E, Rizell S, Marcusson A, Sæle P, Hurmerinta K, Rønning E, Najar Chalien M, Bellardie H, Mooney J, Eyres P, Semb G. Scandcleft randomised trials of primary surgery for unilateral cleft lip and palate: 6. Dental arch relationships in 5 year-olds. *J Plast Surg Hand Surg*. 2017;*51*(1):52-57.

Kara M, Calis M, Kara I, Incebay O, Kulak Kayikci ME, Gunaydin RO, Ozgur F. Does early cleft palate repair make difference? Comparative evaluation of the speech outcomes using objective parameters. *J Craniomaxillofac Surg*.2020;*48*(11):1057-1065.

Karsten A, Marcusson A, Hurmerinta K, Heliövaara A, Küseler A, Skaare P, Bellardie H, Rønning E, Shaw W, Mølsted K, Sæle P, Brinck E, Rizell S, Najal Chalier M, Eyres P, Semb G. Scandcleft randomised trials of primary surgery for unilateral cleft lip and palate: 7. Occlusion in 5 year-olds according to the Huddart and Bodenham index. *J Plast Surg Hand Surg*. 2017;*51*(1):58-63

Küseler A, Mølsted K, Marcusson A, Heliövaara A, Karsten A, Bellardie H, Sæle P, Brinck E, Skaare P, Rizell S, Chalien MN, Mooney J, Eyres P, Shaw W, Semb G. Scandcleft randomized trials of primary surgery for unilateral cleft lip and palate: maxillary growth at eight years of age. *European Journal of Orthodontics*. 2019;42(1):24-29.

Liao YF, Cole TJ, Mars M. (2006). Hard palate repair timing and facial growth in unilateral cleft lip and palate: A longitudinal study. *Cleft Palate Craniofac J*. 2006;*43*(5):547–556

Liao YF, Mars M. Hard Palate Repair Timing and Facial Growth in Cleft Lip and Palate: A Systematic Review. *Cleft Palate Craniofac J.* 2006;43(5):563-570.

Liao YF, Yang IY, Wang R, Yun C, Huang CS. Two-Stage Palate Repair with Delayed Hard Palate Closure Is Related to Favorable Maxillary Growth in Unilateral Cleft Lip and Palate. *Plast Reconstr Surg*.2010;*125*(5):1503-1510.

Lohmander A, Persson C, Willadsen E, Lundeborg I, Alaluusua S, Aukner R, Bau A, Boers M, Bowden M, Davies J, Emborg B, Havstam C, Hayden C, Henningsson G, Holmefjord A, Hölttä E, Kisling-Møller M, Kjøll L, Lundberg M, McAleer E, Nyberg J, Paaso M, Pedersen NH, Rasmussen T, Raisaeter S, Anderson HS, Schops A, Tordal IB, Semb G. Scandcleft randomised trials of primary surgery for unilateral cleft lip and palate: 4. Speech outcomes in 5-year-olds - velopharyngeal competency and hypernasality. *J Plast Surg Hand Surg*. 2017;*51*(1):27-37.

Marrinan EM, Labrie RA, Mulliken JB. Velopharyngeal Function in Nonsyndromic Cleft Palate: Relevance of Surgical Technique, Age at Repair, and Cleft Type. *Cleft Palate Craniofac J*. 1998;*35*(2):95-100.

Pasick CM, Shay PL, Stransky CA, Solot CB, Cohen MA, Jackson OA. Long term speech outcomes following late cleft palate repair using the modified Furlow technique. *Int J Pediatr Otorhinolaryngol*. 2014;*78*(12):2275-2280.

Pereira RMR, Siqueira N, Costa E, Vale DD, Alonso N. Unilateral cleft lip and palate surgical protocols and facial growth outcomes. *J Craniofac Surg*. 2018;*29*(6):1562–1568.

Peterson-Falzone SJ. The relationship between timing of cleft palate surgery and speech outcome: What have we learned., and where do we stand in the 1990s? *Semin Orthod*. 1996;*2*(3):185-191.

Rezaei P, Poorjavad M, Abdali H. Speech outcomes after palatal closure in 3–7-year-old children. *Braz J Otorhinolaryngol*. 2020;S1808-8964(20)30152-X.

Salgado KR, Wendt AR, Fernandes Fagundes NC, Maia LC, Normando D, Leão PB. Early or delayed palatoplasty in complete unilateral cleft lip and palate patients? A systematic review of the effects on maxillary growth. *J Craniomaxillofac Surg.* 2019;47(11):1690–1698.

Shaffer AD, Ford MD, Losee JE, Goldstein J, Costello BJ, Grunwaldt LJ, Jabbour N. The Association Between Age at Palatoplasty and Speech and Language Outcomes in Children With Cleft Palate: An Observational Chart Review Study. *Cleft Palate Craniofac J*. 2020;*57*(2):148-160.

Swanson JW, Mitchell BT, Cohen M, Solot C, Jackson O, Low D, Bartlett SP, Taylor JA. The Effect of Furlow Palatoplasty Timing on Speech Outcomes in Submucous Cleft Palate. *Ann Plast Surg*. 2017;*79*(2):156-161.

Willadsen E. Influence of Timing of Hard Palate Repair in a Two-Stage Procedure on Early Speech Development in Danish Children with Cleft Palate. *Cleft Palate Craniofac J.* 2012;*49*(5):574-595.

Willadsen E, Boers M, Schöps A, Kisling‐Møller M, Nielsen JB, Jørgensen LD, Andersen M, Bolund S, Andersen HS. Influence of timing of delayed hard palate closure on articulation skills in 3‐year‐old Danish children with unilateral cleft lip and palate. *Int J Lang Commun Disord*. 2018;*53*(1):130-143.

Willadsen E, Lohmander A, Persson C, Lundeborg I, Alaluusua S, Aukner R, Bau A, Boers M, Bowden M, Davies J, Emborg B, Havstam C, Hayden C, Henningsson G, Holmefjord A, Hölttä E, Kisling-Møller M, Kjøll L, Lundberg M, McAleer E, Nyberg J, Paaso M, Pederson NH, Rasmussen T, Reisaeter S, Anderson HS, Schops A, Tordal IB, Semb G. Scandcleft randomised trials of primary surgery for unilateral cleft lip and palate: 5. Speech outcomes in 5-year-olds - consonant proficiency and errors. *J Plast Surg Hand Surg*. 2017;*51*(1):38-51.

Williams WN, Seagle MB, Pegoraro-Krook MI, Souza TV, Garla L, Silva ML, Machado Neto JS, Dutka JCR, Nackashi J, Boggs S, Shuster J, Moorhead J, Wharton W, Graciano MIG, Pimentel MC, Feniman M, Piazentin-Penna SHA, Kemker J, Zimmermann MC, Bento-Goncalvez C, Borgo H, Marques IL, Martinelli AP, Jorge JC, Antonelli P, Neves JF, Whitaker ME. (2011). Prospective Clinical Trial Comparing Outcome Measures Between Furlow and von Langenbeck Palatoplasties for UCLP. *Ann Plast Surg*. 2011;*66*(2):154-163.

Xu X, Zheng Q, Lu D, Huang N, Li J, Li S, Wang Y, Shi B. Timing of palate repair affecting growth in complete unilateral cleft lip and palate. *J Craniomaxillofac Surg*. 2012:*40*(8):e358-362.

Yang Y, Li Y, Wu Y, Gu Y, Yin H, Long H, Shi B, Zheng Q. Velopharyngeal Function of Patients With Cleft Palate After Primary Palatoplasty. *J Craniofac Surg*. 2013;*24*(3):923-928.
